# Embolic Events in Infective Endocarditis: A Comprehensive Review

**DOI:** 10.31083/j.rcm2503097

**Published:** 2024-03-07

**Authors:** Gonzalo Cabezon, Paloma Pulido, Javier López Díaz, María de Miguel-Álava, Isidre Vilacosta, David García-Azorin, Adrian Lozano, Andrea Oña, Juan Francisco Arenillas, José-Alberto San Román

**Affiliations:** ^1^Instituto de Ciencias del Corazón (ICICOR), Cardiology Department, Hospital Clínico Universitario, 47003 Valladolid, Spain; ^2^Ciber de Enfermedades Cardiovasculares (CIBER CV), Instituto de investigación Carlos III, 28029 Madrid, Spain; ^3^Instituto Cardiovascular, Hospital Clínico San Carlos, Instituto de Investigación Sanitaria del Hospital Clínico San Carlos (IdSSC), 28040 Madrid, Spain; ^4^Servicio de Neurología del Hospital Clínico Universitario de Valladolid, 47003 Valladolid, Spain

**Keywords:** infective endocarditis, embolic events, stroke, mycotic aneurysm

## Abstract

Infective endocarditis (IE) is a life-threating entity with three main complications: heart failure (HF), uncontrolled infection (UI) and embolic events (EEs). HF and UI are the main indications of cardiac surgery and have been studied thoroughly. On the other hand, much more uncertainty surrounds EEs, which have an abrupt and somewhat unpredictable behaviour. EEs in the setting of IE have unique characteristics that must be explored, such as the potential of hemorrhagic transformation of stroke. Accurately predicting which patients will suffer EEs seems to be pivotal to achieve an optimal management of the disease, but this complex process is still not completely understood. The indication of cardiac surgery in order to prevent EEs in the absence of HF or UI is in question as scientific evidence is controversial and mainly of a retrospective nature. This revision addresses these topics and try to summarize the evidence and recommendations about them.

## 1. Introduction

Despite improvements in the management of infective endocarditis (IE), it remains associated with mortality rates between 20–30% [1]. Morbidity and mortality of IE is mainly driven by the onset of heart failure (HF), uncontrolled infection (UI) and embolic events (EEs) [2, 3].

HF is usually caused by worsening valvular regurgitation. Occasionally, intracardiac fistulae and valvular stenosis are the responsible lesions. HF represents the most frequent complication of IE, occurring in 42 to 60% of native valve IE patients [4, 5, 6]. HF also is the main indication of surgery in 60% of IE cases [7].

UI includes the presence of abscesses, false aneurysms and persisting positive blood cultures despite appropriate antibiotic therapy [2]. Despite high rates of surgery in patients with perivalvular complications [8, 9, 10] mortality remains very high (41%). UI is the second most frequent cause of surgery [7] and the indication associated with worse prognosis [11].

EEs in IE are the result of the migration of vegetation material to any other point of systemic or pulmonary circulation. The two organs most frequently affected by EEs are the brain and the spleen [12]. The incidence of EEs is widely variable [13]. The wide variability in the reported incidence of EEs in IE, largely depends on the inclusion or not of asymptomatic EEs in its calculation [14]. EEs constitute a potentially devastating complication in the course of the disease, especially when the brain is the target organ [14]. Identifying which IE patients are prone to suffer EEs is a challenge and once it has occurred, its optimal management is unknown. While both HF and UI are well established indications of surgery, performing surgery to prevent EEs is more controversial [15]. As a result, AHA and ESC guidelines (American Heart Association and European Society of Cardiology guidelines) indications of surgery for the prevention of EEs are not homogenous [2, 3].

Within this review we summarize and condense the scientific evidence regarding the epidemiology, estimation of risk, prevention of EEs, and management of EEs in IE. We carried out a thorough search in the main databases of medical science to write this review.

## 2. Significance and Importance of Embolic Events in Left-Sided Infective Endocarditis 

**Fig. 1.**
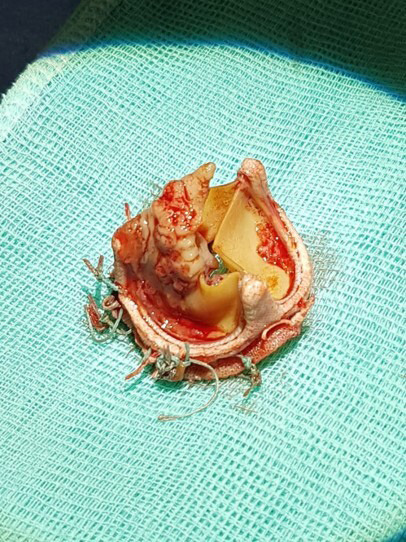
**Vegetation attached to an aortic biologic prosthesis**. Informed consent from the patient for using this image was obtained.

The most frequent sites of embolization in left-sided (LS) IE are the brain and spleen [16]. Nonetheless, it has been taken into account that every place of the systemic circulation is susceptible of being affected, generating process like renal infarct or spondylodiscitis that can be the mode of presentation of the disease. The paradigmatic lesion of IE is the vegetation (Fig. 1), which is defined as an infected mass attached to an endocardial structure or an implanted intracardiac material [1]. Frequently, IE is initiated by an endothelial injury that exposes the subendothelial extracellular matrix, which is capable of activating platelets and causes the formation of a sterile fibrin-platelet clot. Then, microorganisms circulating in the blood adhere to the fibrin-platelet clot to initiate vegetation formation [17]. The embolization of this material is the responsible of EEs in IE.

Regarding clinically significant EEs, incidence varies widely from 13% to 49% [12, 18, 19, 20]. The most feared type of EEs is stroke, which have an incidence of 20–40% in left-sided infective endocarditis (LSIE) episodes [21]. Nevertheless, if we look for asymptomatic EEs, the incidence becomes much higher. When a brain magnetic resonance imaging (MRI) is performed in asymptomatic LSIE patients, the incidence of acute EEs affecting the central nervous system (CNS) was higher than 70% [13]. Incidence of subclinical splenic EEs was studied by Menozzi *et al*. [22], who performed contrast ultrasound in asymptomatic LSIE patients 10-days after the diagnosis of IE, finding that 61% of them showed spleen infarctions. Abdominal EEs were also studied by MRI in consecutive LSIE patients by Iung *et al*. [16]. In this study, up to 34% of patients presented images compatible with abdominal EEs, most of them located in the spleen. In this study, which included 58 patients, 86% had a subclinical EEs diagnosed by brain and abdominal MRI. Thus, evidence suggests that the majority of LSIE patients presents EEs in the course of the disease.

The incidence of clinical EEs is maximal in the days around the diagnosis of LSIE [23], which might be related to the fact that EEs may be the initial manifestation of LSIE and usually leads to its diagnosis. Once LSIE is diagnosed, the distribution of the rate of EEs is not homogeneous along the course of the disease. Embolic risk is highest the day after antibiotic therapy initiation and then its incidence continuously drops within the first two weeks of antibiotic treatment [14, 23, 24]. Nonetheless, this may represent an observation bias as, unavoidably, the infection is still active during the first days of antibiotic treatment.

EEs identification has diagnostic implications, as it can upgrade a possible to a definitive diagnosis of LSIE [25]. In addition to causing variable neurological disability, stroke is an independent adverse prognostic factor for survival [14, 26]. In addition of the inherent mortality of stroke, patients with LSIE who suffer a stroke are more prone to be rejected for cardiac surgery. After suffering a stroke, above 25% of patients do not undergo surgery despite fulfilling guideline criteria for its indication. Mortality rate in this group of patients rises up to 65% [21, 27]. Confirming this finding, Chu *et al*. [28] reported that stroke is independently associated with not undergoing surgery in patients with an indication for intervention.

One of the main complications of an ischemic stroke is its hemorrhagic transformation. This event has especially relevant implications in LSIE, as performing cardiopulmonary bypass is generally contraindicated in the presence of an intracranial hemorrhage. According to American Guidelines of Neurology there are four grades of hemorrhagic transformation: hemorrhagic infarction type I, hemorrhagic infarction type II, parenchymal hemorrhage type I and parenchymal hemorrhage type II [29]. Parenchymal hemorrhage has a potential mass effect and should be aggressively treated.

It has been reported that hemorrhagic transformation occurs more frequently in embolic strokes than in non-embolic strokes [30]. The location, size, and cause of stroke can influence the development of hemorrhagic transformation, and the use of antithrombotic medications, especially anticoagulant and thrombolytic agents, can increase the likelihood of hemorrhagic transformation [31]. Indeed, the rate of transformation in prosthetic valve endocarditis is 42% [32]. Anticoagulation is a well-known risk factor for hemorrhagic transformation and should be use with caution in this setting. Indeed, in specific circumstances such as patients with prosthetic valve IE caused by *Staphylococcus aureus* and a recent central nervous system (CNS) embolic event, it may be considered to withdraw all anticoagulation therapy during the first 2 weeks of antibiotic treatment. Anticoagulation therapy should then be restarted cautiously, and prothrombin time should be monitored carefully [32].

Once a stroke has occurred, its management in the acute phase differs from a stroke in the general population. Reperfusion therapy aims to restore the blood flow to the affected brain tissue. The safety and outcomes of thrombolysis in infective embolic stroke remain a matter of debate. Walker *et al*. [33] reported 18 cases in a retrospective, descriptive case series of IE-related stroke with a mortality rate of 75% for those who received thrombolysis. In another review of 15 case reports with embolic IE, the use of mechanical thrombectomy with or without adjuvant thrombolytics in such situations was reported in 7 cases [34]. The rate of intracranial hemorrhage was 0% in the no thrombolysis vs 50% in the thrombolysis group. Asaithambi *et al*. [35] observed that the incidence of intracranial bleeding in LSIE-related stroke was higher than in the general population (20% vs 6.5%). The high rates of bleeding could partially be explained by the presence of mycotic aneurysms. In summary, patients with IE with acute ischemic stroke are not recommended for thrombolytic therapy [2, 3]. Despite of limited information, mechanical thrombectomy may be a good option, particularly if a large vessel is affected [36]. If performed, the retrieved embolic material should be sent for microbiological analysis.

The clinical management of stroke in the IE patient differs from the general population. According to the American Heart Association/American Stroke Association, alteplase should not be administered to patients with symptoms consistent with IE due to the increased risk of intracranial hemorrhage. These patients should be immediately referred to a tertiary stroke center with 24 h availability of mechanical thrombectomy.

## 3. Mycotic Aneurysms

Mycotic aneurysms are the consequence of small septic embolism to the vasa vasorum or the intraluminal space. An acute inflammatory cascade altering the vessel wall is brought about and arterial dilatation occurs [37]. Mycotic aneurysms are specific lesions of LSIE that are present in 2–4% of LSIE patients [38] and are usually asymptomatic if unruptured. Every arterial vessel can be affected by a mycotic aneurysm, but most of them are located in cerebral arteries, especially in branches of the middle cerebral artery [39]. The rupture of a mycotic aneurysm causes intracranial, intraventricular or subarachnoid hemorrhages. Among neurological complications in LSIE patients, mycotic aneurysms represent approximately a 5% [40] and its rupture entails mortality rates of approximately 35–40% [41]. The true incidence of mycotic aneurysms when performing cerebral angiography irrespective of symptoms is around 9% [38].

**Fig. 2.**
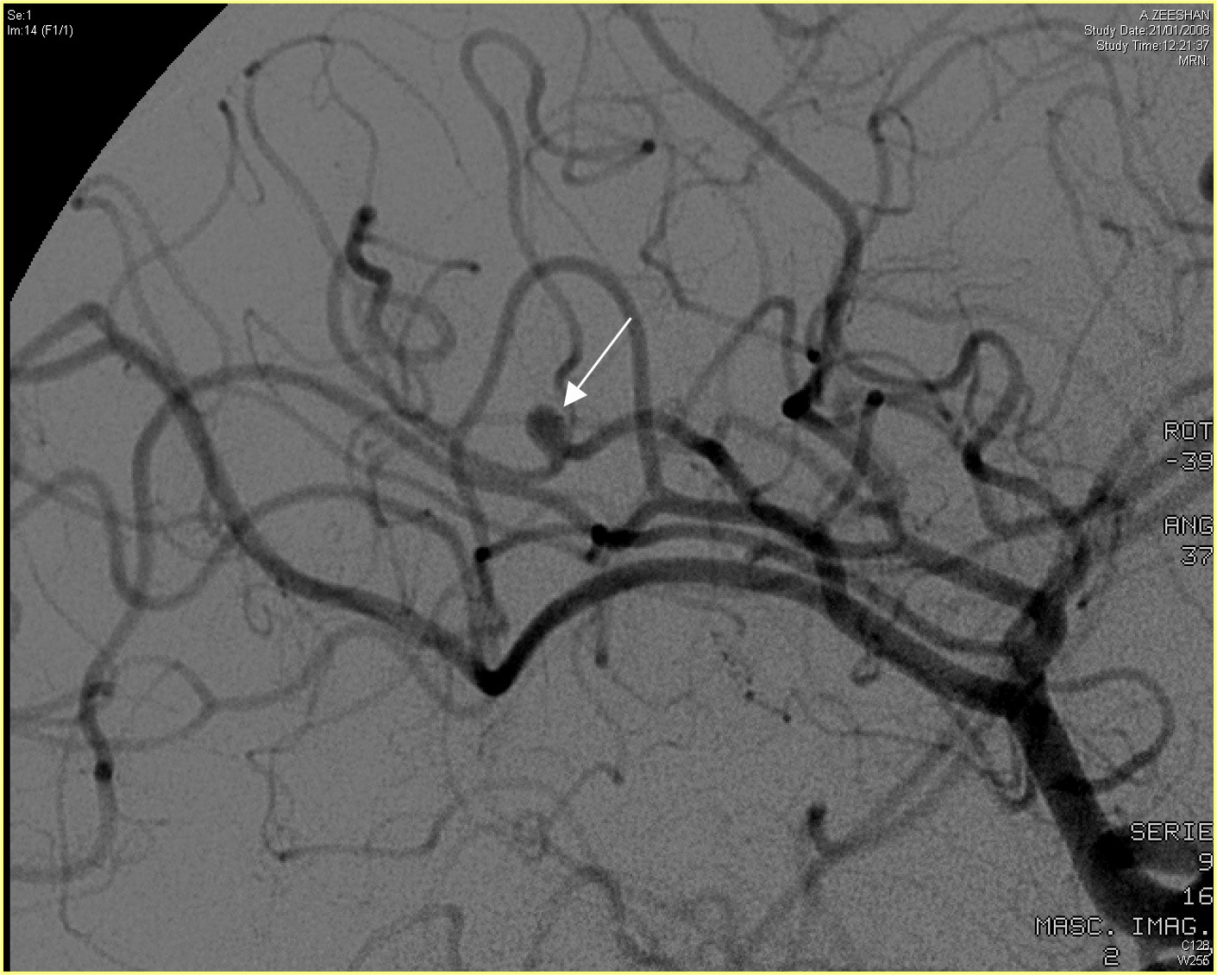
**Cerebral arteriography showing the presence of a mycotic aneurysm**. Informed consent from the patient for using this image was obtained.

The presence of a mycotic aneurysm in LSIE should be suspected when an intracranial bleeding occurs. Given the low sensitivity of both computed tomography and MRI for small aneurysms (57% and 35% respectively) [42], intraarterial cerebral angiography (Fig. 2) remains the gold standard test and high index of suspicion should prompt its realization in the setting of an intracranial bleeding [39].

Evidence regarding management of mycotic aneurysms is limited to retrospective studies [43, 44]. Antibiotic treatment is known to effectively reduce the size of mycotic aneurysms and it is recommended in all patients. In the presence of an intracranial hemorrhage, neurosurgical treatment is recommended [45]. Endovascular techniques provide high occlusion rate and low rate of procedure-related complications [37] and are preferred over microsurgical approach, especially if the patient is undergoing early cardiac surgery, as cardiopulmonary bypass can be performed even in the same day as endovascular treatment [42, 43]. On the contrary, open surgical approach entails waiting 2 weeks for the cardiopulmonary bypass to be safe. There is evidence that patients who undergo cardiac surgery and presented preoperative mycotic aneurysms suffer a higher incidence of intracranial hemorrhage in the hospital and in the long term [46].

## 4. Timing of Surgery after Ischemic and Hemorrhagic Transformation

The optimal timing for surgical intervention in patients who have already had a stroke is contentious, with several older studies suggesting poor outcomes from early surgery [21]. The high level of anticoagulation for cardiopulmonary bypass and hypotension during surgery theoretically worsens cerebral ischemia and favors hemorrhagic transformation [47]. As stated before, there are four grades of severity of hemorrhagic transformation of an ischemic stroke [29] and it has been to be considered for an adequate management.

Early surgery was found to be associated with a nonsignificant increase in hospital mortality when compared with patients operated on later (>7 days) after stroke, suggesting that early cardiac surgery after ischemic stroke is not contraindicated and can be performed without delay with acceptable operative and longer-term survival when indications for surgery are present.

Both ESC and AHA guidelines [2, 3] recommend delaying cardiac surgery 4 weeks if a major ischemic stroke or intracranial hemorrhage occurs. Nonetheless, Barsic *et al*. [48] showed that early surgery after ischemic stroke can be performed without delay with acceptable operative and longer-term survival when other indications for surgery are present. Subsequently, ESC guidelines state a IB indication for performing surgery without delay after an ischemic stroke if HF, UI or persistent high embolic risk is present once intracranial hemorrhage is discarded by computed tomography [2].

## 5. Stratifying the Risk of Embolic Events in LSIE Patients 

Identifying the patients that are at the highest risk of suffering EEs is of utmost importance in the management of the disease. Despite many factors have been proposed to have an influence in embolic risk, the most recognized and robust parameter to predict the embolic risk of an LSIE patient is vegetation size. Several classic [49, 50] and contemporary [51, 52] studies have observed that larger vegetations are associated with increased rates of EEs. Mohananey *et al*. [53] conducted a meta-analysis of 21 studies published for 4 decades and concluded that vegetation length >10 mm is associated with EEs. Interestingly, vegetation size has also been correlated to a correct IE diagnosis, being the optimal cut-off value of 11.5 mm [54]. As a result, European and American guidelines base their indications of surgery in vegetation size [1, 2] and some randomized [55] and observational [56] studies have been designed putting vegetation size in a central position for its design.

Nonetheless, is worth emphasizing the limitations of vegetation size to accurately predict EEs for technical and clinical reasons.

Theoretically, vegetation volume would be a more reliable predictor of EEs than vegetation length. However, vegetation size is measured considering just the maximal length of a vegetation [57]. The arrival of 3-dimensional (3D) transesophageal echocardiography (TEE) allows more accurate characterization of vegetation compared to 2-dimensional (2D) TEE. Indeed, the cutoff points best related to an increased risk for EEs during LSIE are >16.4 mm for 3D TEE and 9.5 mm for 2D TEE [58]. Another limitation of this parameter is reproducibility. Recently, a high 2D TEE interobserver variability of vegetation size measurement has been found; remarkably, the indication of surgery would have changed in up to 43% of patients depending on which operator measured the vegetation [59].

Vegetation size is not the only image variable that has been linked to EEs. Mobile vegetations showed higher rates of EEs in this meta-analysis which employed the data from 6 studies [60]. Another well-established variable associated with EEs in LSIE is the location of the vegetation [24]. Mitral location of vegetation, particularly in the anterior leaflet [61], entails higher embolic risk than aortic location [60]. Also, vegetation morphology has been associated with EEs, as filiform and raceme-shaped vegetations present higher rates of EEs than sessile vegetations [62]. Likewise, it has been described that pulmonary embolism was more frequent in globular vegetations than in filiform or sessile vegetations in device-related IE [63]. Finally, a high level of local inflammation assessed by fluorodeoxyglucose (F-FDG) Positron Emission Tomography in both native and prosthetic valve LSIE has been associated with high rates of EEs [64].

Among clinical variables, probably the most relevant one is the initiation and time of appropriate antibiotic treatment. It is well-known that antibiotic treatment reduces effectively the risk of EEs as it helps in controlling active infection [24]. On the other hand, vegetation stability can be influenced in the early phase of antimicrobial therapy as it modifies vegetation size [65]. Thus, during the first days of antibiotic therapy the embolic risk is the highest and it decreases over time. Indeed, the embolic risk of a vegetation is 10–20 times higher the day after antibiotic therapy initiation compared to 2 weeks later [23].

Embolism rates are influenced by the responsible microorganism causing LSIE. *S. aureus* has been associated with poor prognosis and it has been proposed as a major risk factor for EEs [24], with rates of EEs of approximately 35% depending on the series studied [66]. *S. aureus* has been identified as an independent risk factor for EEs in LSIE in a meta-analysis of 19 studies [60]. Also, other microorganisms, like *Streptococcus gallolyticus * [67] or fungal [68, 69] LSIE have been suggested as independent risk factors for EEs.

Given the multivariable influence of EEs risk in LSIE, Hubert *et al*. [70] developed predictive models to assess embolic risk. The result was a 6-month risk calculator using six variables: age, diabetes, atrial fibrillation, embolism before antibiotics, vegetation length and *S. aureus* infection [70]. This calculator has been validated in Japanese [71] and Filipino [72] population and might be a useful tool to predict EEs in LSIE.

To sum up, vegetation size is the most important independent predictor of new EEs and the one which guides the indication of surgery. However, its measurement entails some difficulties and there are plenty of other variables influencing embolic risk, so it is far from being a perfect parameter to predict EEs.

## 6. What do the Guidelines Recommend? 

Surgical indications to prevent EEs can be divided into secondary and primary prevention. Regarding secondary prevention, both ESC and AHA guidelines [2, 3] recommend surgery to prevent recurrent EEs in patients who suffer EEs despite appropriate antibiotic therapy and have persistent vegetations. ESC guidelines vegetation size threshold is >10 mm and no length threshold is specified by AHA guidelines. The level of recommendation is I in ESC and IIa in AHA guidelines.

Regarding the recommendation of surgery in primary prevention of EEs, 2023 ESC guidelines state that surgery may be considered in patients with a vegetation >10 mm and low surgical risk. Importantly, it is specified that surgery should be urgent, defined as within the 3–5 days after the decision. On the other hand, according to AHA guidelines surgery may be considered at any time during the antibiotic treatment period. In both guidelines the level of recommendation is IIb.

Of note, surgical indications in primary prevention of EEs have changed from the last ESC guidelines (Table 1). In 2015 ESC guidelines [73] surgery was recommended in primary prevention of EEs when vegetation size was >30 mm (IIa) and >15 mm (IIb). A third indication was vegetation >10 mm with severe valve stenosis/regurgitation and low surgical risk (IIa). In the 2023 ESC guidelines these indications have been condensed in one: vegetation >10 mm and low surgical risk (IIb), downgrading any possible indication of surgery in primary prevention of EEs to IIb and restricting it to low surgical risk patients.

**Table 1.** **Indications of surgery as primary prevention of embolism in LSIE**.

ESC guidelines 2015	ESC guidelines 2023
Aortic or mitral NVE or PVE	
Surgery should be considered in primary prevention of EEs when vegetation size >30 mm (class IIa, level of evidence B).	Surgery should be considered in patients with vegetation >10 mm and low surgical risk (class IIb, level of evidence B).
Surgery should be considered in primary prevention of EEs when vegetation size >15 mm (class IIb, level of evidence C).	
Surgery should be considered if there is a vegetation >10 mm and it is associated with severe valve stenosis/regurgitation in patients with low surgical risk (class IIa, level of evidence B).	

EEs, embolic events; LSIE, left-sided infective endocarditis; NVE, native valve endocarditis; PVE, prosthetic valve endocarditis; ESC guidelines, European Society of Cardiology guidelines.

## 7. Evidence Supporting Surgery to Prevent Embolic Events in LSIE

There is evidence that large vegetations (>10 mm), are associated with higher rates of EEs and increased mortality [53, 74, 75, 76]. However, it is unclear if performing surgery in these patients improves survival, especially if HF and UI are absent.

The main observational study that specifically aimed to evaluate the influence of surgery in LSIE patients with large vegetations was performed by Fosbøl *et al*. [56]. In a cohort of 1006 patients, those with vegetations >10 mm presented higher mortality rates than those with small vegetations. After propensity adjustment, the association with higher mortality persisted only in those patients with large vegetations who were managed medically rather than surgically.

Vegetation size seems to identify patients with worse prognosis, as they found that large vegetations were associated with more IE-related complications, including embolic events, HF, paravalvular complications, valve perforation and persistent bacteremia. Therefore, the lower mortality rates observed in patients with large vegetations who underwent surgery may be related to improvement in several of these prognostic complications, including treatment of HF and UI, rather than reducing EEs.

Only one randomized controlled trial has been carried out in patients with LSIE to assess the effect of surgery in preventing EEs [55]. In this work, Kang *et al*. [55] included 76 patients with a vegetation >10 mm and severe mitral or aortic valve disease. Key exclusion criteria were moderate-to-severe HF, abscess, destructive penetrating lesions requiring urgent surgery, and prosthetic and fungal endocarditis. Patients were randomly assigned to early surgery or conventional treatment. Early surgery was defined as surgery performed within 48 hours after randomization. The composite of in-hospital death or clinical EEs 6 weeks after randomization was the primary end point. A secondary end point was death or clinical EEs at 6 months of follow-up. Primary and secondary end points occurred more frequently in the conventional treatment group driven by EEs rates. However, in-hospital and 6-month mortality was not different between groups.

This randomized controlled trial was a single-center study, all of the patients had severe valve disease by inclusion protocol and surgical mortality was strikingly low. On the other hand, cross-over was frequent, as the majority of patients (77%) of the conventional therapy group underwent surgery during the initial hospitalization, potentially diluting the beneficial effect in survival of surgery. Thus, this study left partially unanswered whether surgery should be performed in patients with large vegetations and no other indications of surgery.

The ASTERIX trial [NCT05061355] is currently randomizing patients with LSIE and large vegetations (≥10 mm) without other Class 1 indication to early surgery or conventional treatment and will assess overall mortality, stroke, or another systemic embolism.

## 8. Embolic Events in Right-Sided Infective Endocarditis (RSIE)

IE affecting the right heart chambers and valves accounts for 10–15% of all IE cases [77]. Compared with LSIE, much less information on right-sided infective endocarditis (RSIE) is available. RSIE is often associated with intravenous drug use and intracardiac devices. These groups of patients present low mortality rates compared with LSIE [78]. However, in the absence of drug use and intracardiac device, RSIE may occur. Recently, this type of RSIE has been named “three noes IE” (no LSIE, no drug use, no intracardiac device) [79]. In this group of patients, mortality is comparable to LSIE patients [80].

The main complications in RSIE are valvular regurgitation, septic pulmonary embolism, and pulmonary abscess [81]. Mortality in RSIE is driven by right HF and UI [82]. However, EEs can disseminate the infection to the lungs and worsen right heart failure due to increase in pulmonary pressures [74]. Lastly, systemic paradoxical embolism is rare, but could occur in the presence of an intracardiac shunt. EEs in RSIE are common and sometimes subclinical. Indeed, Rizzi *et al*. [75] reported RSIE as a risk factor for EEs.

As in LSIE, vegetation size is the variable most tightly associated with EEs in RSIE. Abubakar *et al*. [83] reported that the risk of septic pulmonary embolism is approximately 34% to 55% in patients with vegetations >1 cm. Galzerano *et al*. [84] identified vegetation size >15 mm as the strongest predictor of EEs in RSIE.

Management of RSIE-related EEs is based in antibiotic treatment [82] and similarly to LSIE reduces the incidence of EEs as the infection is controlled. Hemodynamic instability due to RSIE-related pulmonary embolism is a rarity, but there are case reports of pulmonary endarterectomy in this setting [85, 86].

To prevent EEs, cardiac surgery is recommended when persisting large residual vegetations (>20 mm) after recurrent septic pulmonary emboli [2]. Recently, the extraction of large vegetation has been described using percutaneous extracorporeal circuitry for aspiration [87]. Patel *et al*. [88] reported the use of percutaneous aspiration device to reduce the incidence of septic pulmonary emboli. Percutaneous aspiration recommendation is reserved to patients with high surgical risk [2].

## 9. Future Directions

One of the main challenges in LSIE management is to accurately predict the individual embolic risk. Despite being the most robust parameter, vegetation size is not the only variable that predicts EEs. Artificial intelligence may help to develop reliable scores to accurately predict EEs and therefore identify those patients that should undergo cardiac surgery.

A non-invasive procedure to reduce embolic risk would be a great breakthrough in LSIE management, especially in non-operable patients. As stated before, percutaneous vegetation aspiration systems are being increasingly used in RSIE [87, 88]. Percutaneous aspiration of vegetations in LSIE is much more challenging than in RSIE mainly due to vascular access and embolic risk. Reaching LSIE vegetations implies a retro aortic or across the interatrial septum access. Besides, dropping vegetation material into the bloodstream could precipitate a systemic embolism, especially to the brain. Nonetheless, there are reports of successful percutaneous aspiration of mitral valve vegetations [89] using brain protection devices to avoid stroke. Further randomized controlled studies are warranted to test these procedural techniques and determine safety and outcomes of percutaneous aspiration in LSIE.

## 10. Conclusions

EEs in LSIE can be devastating and entail prognostic implications. EEs prediction is still imperfect and scientific community should keep improving the stratification of patient embolic risk in order to optimize its management. Evidence supporting cardiac surgery performance in order to avoid EEs is controversial and should be taken cautiously. Randomized controlled trials are warranted to further clarify the benefit of performing surgery in the absence of HF or UI. We emphasized the formation of Endocarditis Team Committees composed by infectious disease specialist, neurologists, cardiac surgeons and cardiologists in order to deal with this systemic disease.
